# The paradigm shift for drug delivery systems for oral and maxillofacial implants

**DOI:** 10.1080/10717544.2018.1477855

**Published:** 2018-07-03

**Authors:** Rafal Pokrowiecki

**Affiliations:** a Department of Otolaryngology and Ophtalmology, Prof. Stanislaw Popowski Voivoid Children Hospital Department of Head and Neck Surgery – Maxillofacial Surgery, Zołnierska, Olsztyn, Poland;; b Private Dental Practice, Poland

**Keywords:** Drug delivery systems, implants, oral cavity, infections

## Abstract

Along with the development of nanotechnological strategies for biomaterials associated with the prevention of infections, a myriad of clinically unproven techniques have been described to date. In this work, the aim was to perform a critical analysis of the literature available concerning antibacterial biomaterials for oral implantology and to provide a practical derivation for such a purpose. As anti-adhesive strategies may affect osseointegration, they should no longer be recommended for inclusion in this class of biomaterials, despite promising results in biomedical engineering for other, non-bone load bearing organs. Targeted, antibacterial drug delivery is most likely desirable in the case of intraosseous implants. Interfering factors such as the oral cavity environment, saliva, the bacterial microbiome, as well as, the characteristics of the alveolar mucosa and peri-implant space must be taken into account when calculating the local pharmacokinetics for antibacterial coatings. Effective release is crucial for tailoring antibacterial implant longevity providing minimal inhibitory concentration (MIC) for the desired amount of time, which for oral implants, should be at least the cumulative time for the osseointegration period and functional loading period within the tissues. These parameters may differ between the implant type and its anatomical site. Also, the functional drug concentration in the peri-implant space should be calculated as the amount of the drug released from the implant surface including the concentration of the drug inactivated by biological fluids of the peri-implant space or saliva flow throughout the effective release time.

## Introduction

Infections associated with implantable devices, also known as biomaterial-associated infections (BAIs) pose a serious problem in contemporary regenerative medicine and traumatology. In recent years, the number of procedures which make use of different biomaterials in oral and maxillofacial surgery (OMFS) have significantly increased. This is mainly due to an increase in the number of accidents of major magnitude which require diversified materials and techniques to restore the esthetic and functional integrity of the craniofacial area. Despite efforts being made in bioengineering to improve the biocompatibility of metallic biomaterials, which constitute a major part of reconstructive surgery, the problem of bacterial settlement and infection development still poses a serious threat to treatment outcome (Goodman et al., 2013). Therefore, BAIs are regarded as a significant burden on the patient and healthcare system, as they increase the number of clinic visits required during a patient’s course of treatment. BAIs result in delayed healing and nonunions, scaring, an unsatisfying esthetic outcome, and a worsening of the clinical prognosis in general (Kazmers et al., 2016; Rams, 2017). The treatment of such infections is also frequently complicated due to the increasing global resistance of bacteria to commonly used antibiotics (Gallo et al., 2014). With the development of nanotechnology, a new approach to biomedical devices has arisen through the application of smart biomaterials, which in theory, are supposed to exhibit a multifactorial effect on the surrounding biological environment through activity at a submicron-level (Arciola et al., 2015). Among these factors, induced biocompatibility, regenerative potential and antibacterial properties are the expected outcomes of properly tailoring the material’s chemical structure, surface properties, and coatings. In this critical review, the requirements for new antibacterial nano-biomaterials for the purposes of oral and maxillofacial surgery will be outlined.

## Intra- and extra-oral infections around implantable devices: fade in

In order to better understand the clinical need for antimicrobial implants for the head and neck, one must acquire the types of implants commonly used in this anatomical area. The new classification scheme presented serves this purpose (Table 1). Oral and maxillofacial (OMF) implants are anchored within the facial or skull bones and are introduced into the body either though an intra- or extra-oral approach or sometimes both in the case of large scale reconstructions of the orofacial region.

**Table 1.** New classification of oral and maxillofacial (OMF) implants on the basis of their anatomical area and expected functionality.

Type	Class	Subtype	Time of full integration with OMF bone tissue (*Tint*)	Time of functional loading	Expected functionality period *(Tfun)*	Examples
Partially external (E)	Permucosal (M)	Permanent (p)^b^	3–6 months post. op.	3–6 months post. op.^a^	Minimum 10 years	Dental implants
		Temporary (t)		At a day of implantation up to 1 month	Several weeks/months	Orthodontic mini-implants Intra-maxillary fixation (IMF) screws, distractors
	Percutaneous (C)	Permanent (p)		At a day of implantation up to 1 month	Minimum 10 years	Epithesis anchor
		Temporary (t)			Several weeks/months	Skull and facial distractors, pins, wires.
Internal (I)	Permucosal (M)	Temporary (t)		At a day of implantation	Several weeks/months	Mini- and microplates
	Percutaneous (C)	Temporary (t)		At a day of implantation	Several weeks/months	Mini- and microplates

a Some implants are loaded soon after the surgery, e.g. miniplates for fracture fixation, dental implants, IMF screws, orthodontic screws and others, depending on the protocol and surgeon’s preferences.

b Current standards require minimum 10-year undisturbed functionality of a dental implant in the oral cavity. The same for epithesis-bearing implants.

The period of time needed for biological bonding of an implant within bony tissue (described as osseointegration) (abbreviated here as *time of integration: Tint*) is usually estimated at 3–6 months. The functional loading of an implant may be performed either soon after a surgery or after osseointegration has been established. For example, dental implants are usually loaded after 3–6 months, whereas, orthodontic mini-implants are loaded within 2 weeks to 1 month postoperatively. Miniplates for fracture fixation are loaded soon after the surgery. Permucosal bone distractors are activated from one postoperative day up to one week after device implantation and left for up to 3 months after osteogenesis is completed (Table 1). Consequently, the desired period of implant functionality (abbreviated here as *Tfun*) may differ between implant types. In some cases, implant exploitation begins just after a surgery, in other cases it must be delayed until the device is more stable within the bone, in order to fully comprehend the loading and shear forces. Therefore, the *Tint/Tfun* ratio is strictly related to the manufacturer’s requirements as well as treatment expectations and will have a direct impact on bacterial biofilm formation, susceptibility to infection, treatment possibilities, and prognosis.

**Figure 1.**
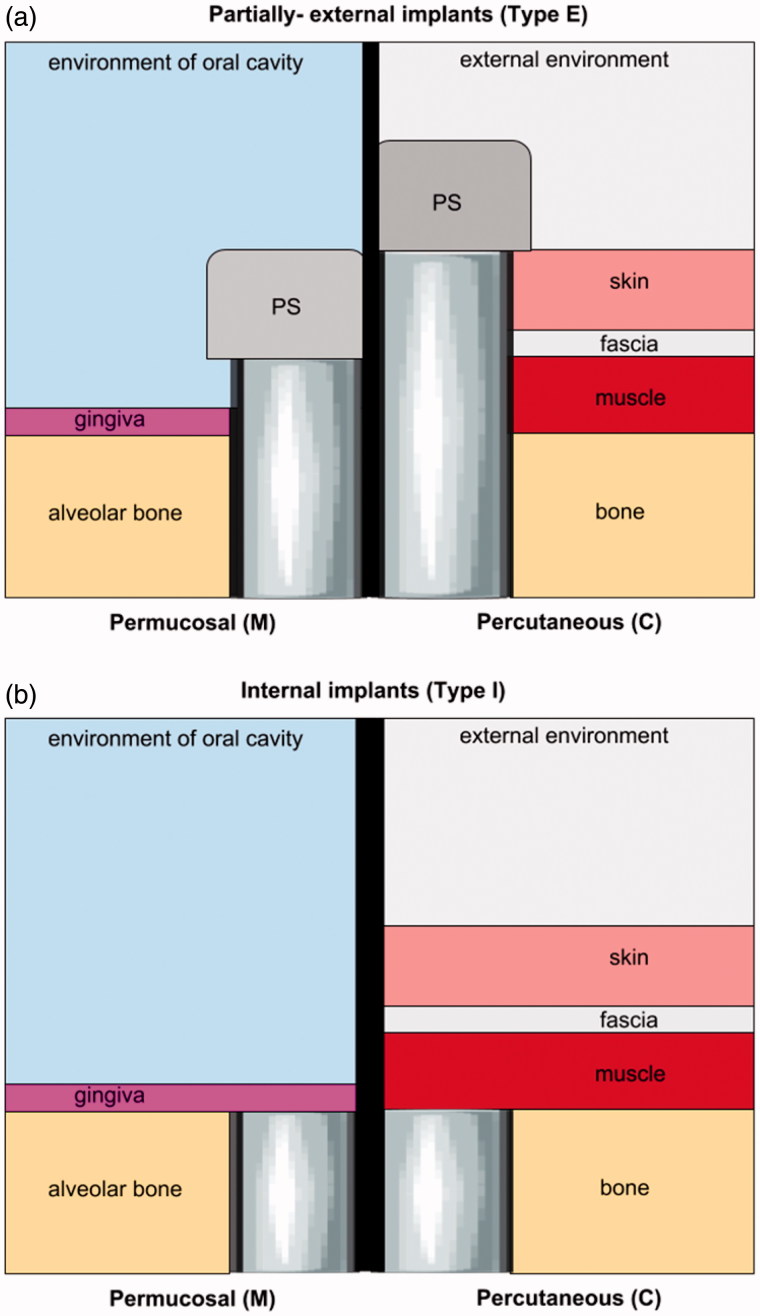
An illustration of partially-external (Type E) (a) and internal (Type I) (b) implants. Type E permucosal implants (m) are embedded in alveolar bone and, by piercing gingiva are exposed to the environment of the oral cavity to reach the prosthetic superstructure (PS). Otherwise, percutaneous implants that are fixed in the bone, pierce different soft tissues (muscle, fascia, skin, and sometimes fat) until they reach the outer environment and PS. Consequently, each Type E implant has its bone-embedded part and soft tissues-piercing part* which is constantly under increased risk of bacterial infiltration. Conversely, internal implants (Type I) are fully covered with a soft tissue seal and remain separated from the external environment. **The ratio bone: soft tissues, their cytoarchitectonic, histological origin, and anatomical site influence susceptibility to infection.

Biofilm formation on medical devices, regardless of the environment, follows a four-step process: (1) initial attachment of bacterial cells; (2) cell aggregation and accumulation in multiple cell layers; (3) biofilm maturation and (4) detachment of cells from the biofilm into a planktonic state, spreading along the material’s surface to initiate a new cycle of biofilm formation (Møller et al., 2002; Ferreira & Marais, 2012; Ramasamy & Lee, 2016). The whole process is preceded by a biofouling effect, which is surface preconditioning and the adsorption of unwanted macromolecules that leads to the formation of a layer favorable for bacteria to attach to (Maruyama et al., 2015). The presence of environment-specific biological fluids and the anatomical location of the implant influence the dynamics of the biofouling effect and subsequent bacterial biofilm neo-genesis. It also influences microbial diversity, as well as, its virulence factors which have an impact on the biology, course, and nature of peri-implant infection (Figure 1).

**Figure 2.**
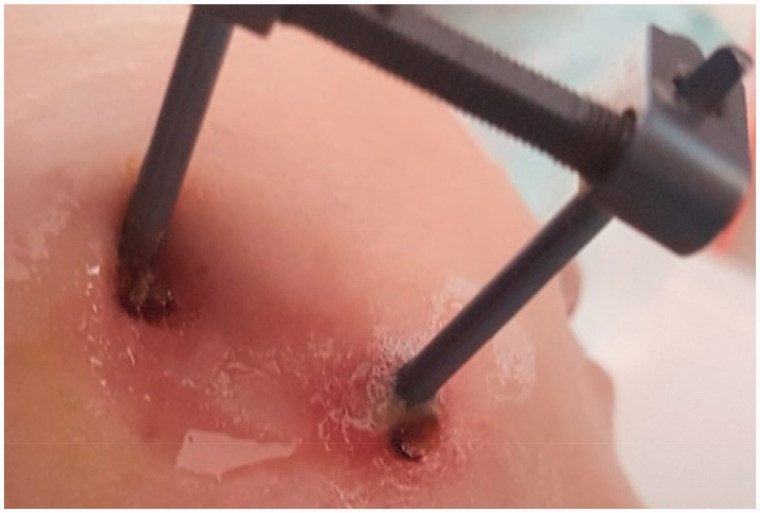
Clinical manifestation of pin site infection in the area of the head and neck around an ECt implant where redness, swelling, and pus discharge may be observed.

Therefore, extraoral infections significantly differ from intraoral ones, and require completely separate approaches to prevention, peri- and/or postoperative antibiotic therapy, and in the case of infection development; lavage, drainage, local and/or systemic antibiotic therapy, or removal. Extraoral BAIs are mainly associated with transcutaneous biomaterials used in the area of the head and neck. Among these, one can distinguish temporary materials such as distractor pins, guideways or wires used for facial distraction osteogenesis (ECt implant group) (Table 1 and Figure 1(a)). Another group of extraoral biomaterials used in OMFS are permanent, transcutaneous implants (ECp implant group) used for the fixation of prosthetic devices such as the ear, eye, or other epithesis, used in patients who underwent oncological ablative surgery procedures (Table 1 and Figure 1(a)). Both, ECt and ECp implants are under an increased risk of staphylococcal and streptococcal bacterial infection, described as ‘pin site infections’ (PSI) or ‘pin tract infections’ (PTI) (Figure 2) (Kazmers et al., 2016) which may lead to device instability, osteomyelitis, and the need for removal.

It is estimated, that PSIs constitute the majority of complications associated with transcutaneous implants in the OMFS area; however, the comprehensive classification of such infections is still lacking. The susceptibility to infection has been linked to patient-related general factors such as diabetes, rheumatoid arthritis, and other collagen vascular diseases and smoking. The patient-related local factors which contribute to infection are: an extended duration of pin fixation and their near proximity to joints and sites with greater soft tissue thickness over bone (movable and more soft tissues around a pin) (Costalonga & Herzberg, 2014). The surgery-related factors influencing the incidence rate of peri-implant infections are: surgery and pin insertion technique, as well as tissue handling. Apart from antibiotic prophylaxis, the application of cleansing solutions or dressings there is no consensus regarding optimal PSI prevention and treatment protocols (Camps-Font et al., 2015; Kazmers et al., 2016).

The oral cavity is a distinctive ecosystem which significantly differs from all others, across the human body. This is due to the presence of protein and sugar-rich saliva and bacteria which covers all surfaces in the mouth. Intraoral partially external (Em) implants are constantly exposed to bacterial attachment, regardless of the hygienic oral measures due to the biofouling effect of saliva’s constituents. Among Em implants, one may include: dental implants, orthodontic plates and mini-implants, distractors and intra-maxillary fixation screws (Table 1).

**Figure 3.**
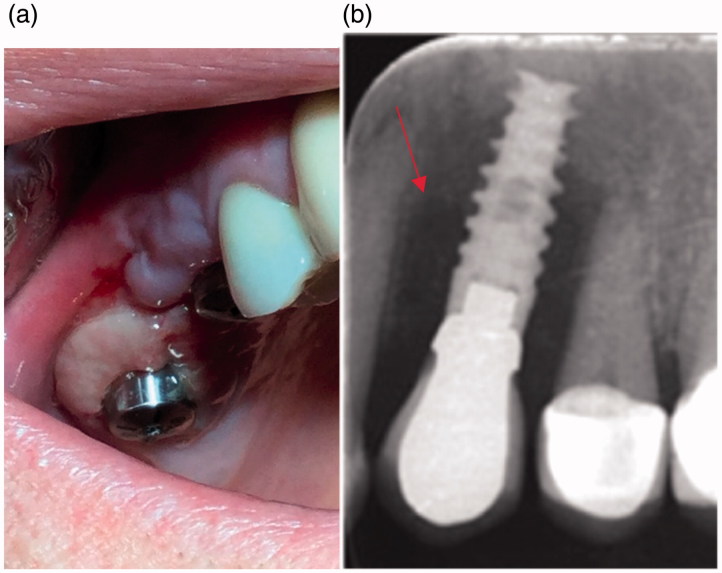
Clinical symptoms of chronic infection around implant healing abutments with fibrotic, inflammatory overgrowth of the gingiva (a) and radiological findings around dental implant bone resorption (red arrow) (b).

The etiology of peri-implant diseases in the oral cavity is complex and includes general and local patient-related factors but is also influenced by material properties, saliva components, and the more frequently postulated human oral microbiome which may differ between individuals (Costello et al., 2012; Graham & Cady, 2014; Romanò et al., 2015). The initial stage of infection is restricted to the peri-implant mucosa, known as peri-implant mucositis (PIM). At an early stage it may be clinically unnoticeable, but redness, excessive swallowing and bleeding from the soft-tissue collar is a pathognomonic sign of the disease. Advanced infection described as peri-implantitis (PI) which is mostly driven by mixed anaerobic microbiota (*Porphyromoas gingivalis, Tanarella forsythia, Treponema denticola*) (Schricker et al., 2012) affects the bone, resulting in its resorption, pus discharge and implant loosening, which is an indication for implant removal (Figure 3). Peri-implant infections in the area of the head and neck may occur weeks to years post-implantation, frequently with a course of latent infections (Hsiao et al., 2014).

### Current approaches to infection control around implants and their potential for the head and neck area

Experimental studies introduced several approaches to biofilm reduction around biomedical implants (Scardino et al., 2009). According to the classification given by Gallo et al. (2014), antibacterial implants may be divided into three groups: for the prevention of adhesion and adsorption, antibacterial, and multi-functional (Arciola et al., 2015). The type of implant and its coating must be adapted to the clinical purpose and anatomic site. Also, antibacterial properties must be weighted with the biological and biomechanical properties of the biomaterial, to fully appreciate its potential. The requirements for an ideal antibacterial biomaterial were described further by Romano et al. (2015). The biomaterial should exhibit a beneficial effect on bone healing, no toxicity and prolonged antibacterial activity without contributing to resistance growth. Additionally, it should exhibit the appropriate biomechanical properties such as adjusting stiffness, elastic modulus and resistance to press-fit insertion, as well as being easy to handle and costing an acceptable amount (Feng et al., 2015).

### Anti-adhesive biomaterials and passive surface finishing – the need to sift the wheat from the chaff

The aim of anti-adhesive biomaterials is to prevent peri-implant infection through the inhibition of the process of bacterial biofilm formation at the very beginning. This approach has been discussed recently, mainly through nanoscale modification of the surface features in order to selectively drive or inhibit biofouling which precedes biofilm formation (Maruyama et al., 2015; Slepicka et al., 2015). This may be achieved through the modification of the physico-chemistry of the surface layer of a biomaterial via its wettability (Ca), roughness (Ra), chemical structure, organization of topography, and orientation of the surface nanofeatures (Koc et al., 2008; Rivardo et al., 2009; Bazaka et al., 2015). These factors are inter-connected, and changes in the value of one may affect all of the others. However, it has been noted that at a nanoscale level, the roughness value is the most important factor for antifouling properties (Bazaka et al., 2015).

Roughness (Ra), describes all surface irregularities from the macro to the submicron scale, it has been extensively evaluated with regard to tissue healing and biofilm formation. From the macroscopic perspective it is generally recognized that every crack, pit, thread and other features may serve as a favorable niche for bacterial leakage, settlement, and biofilm formation. This is certainly the case with dental implants and their shape, threads, pits, cracks, etc. In recent years, it has been proven that roughening the surface did not significantly improve implant interconnection with bone, but rather, incomparably increase infection severity. This was observed especially in those implants with micro-structured hydroxyapatite coatings which would detach from the surface after implant press-fitting, irritate surrounding tissues as a cumulative bacterial biofilm and induce inflammation. The studies of surface roughness at a nano-structure level have been extensively evaluated and it was postulated that it may be possible to directly influence cells and tissues response through mimicking the irregularities of the extracellular matrix (ECM) (undulations, bends, branches) as nano-scale roughness corresponds directly with the protein dimension (Parra et al., 2015; Pechook et al., 2015). Although nanostructured implants do indeed exhibit faster osseointegration, probably through a more active surface, the problem of BAIs is still unsolved. An attempt to obtain an antiadhesive nanosurface has been described in several papers. Through the appropriate modification of the nano-surface chemistry and roughness, bacterial repulsion may be achieved either by steric repulsion, low surface energy, or electrostatic repulsion (Pogodin et al., 2013). To achieve this effect, different nano-coatings were investigated *in vitro* such as: poly(ethylene glycol), poly(methyl oxazoline), polyacrylamide, zwitterionic poly(carboxybetaine methacrylate) and poly(sulfobetaine methacrylate), albumins (Krachler & Orth, 2013), self-assembled monolayers, nanowires, polymer brushes (Pechook et al., 2015), graphene (Wojnicz & Jankowski, 2007) and others (Laureti et al., 2013; Feng et al., 2015). More recently, bio-inspired nano-topographies based on taro leaves, slippery surfaces or with super-hydrophobic paraffin or fluorinated wax crystals were evaluated as well (Dukhin & Labib, 2012). Pogodin et al. (2013) developed the biophysical model of the interaction of bacterial cells with hydrophobic nano-pillar structures of cicada wings and concluded that certain nano-patterns may directly injure the cell membrane in the regions suspended between the pillars (Park, 2014). In a study by Feng et al. (2015), it was shown that with a given nano-porous topography, it is possible to minimize bacterial attachment by repulsive forces, electrostatic, and acid–base forces originating from pores (Rivardo et al., 2009). The other, experimental approach to an anti-adhesive prevention strategy was through the disruption of the bacterial surface receptor biogenesis (Lyndon et al., 2014). Several studies have described the efficacy of sub-inhibitory concentrations of certain antibiotics (fluoroquinolone ciprofloxacine and the aminoglycoside amikacin) that altered the physicochemical properties of the bacterial surface and bacterial adhesion (Gao et al., 2011; Gimeno et al., 2015). However, it is currently well recognized that sub-inhibitory concentrations of antibiotics have a significant effect on bacterial mutation rates and horizontal gene transfer which contribute to the emergence and, as a consequence increase the capacity of bacteria to resist higher doses of antibiotics (Gulati et al., 2012).

It was shown that bacteria are more likely to adhere to hydrophobic surfaces but there is no well-established pattern for the adhesion profile (Goodman et al., 2013), especially as intra-individual oral biofilms may be composed of over 700 species (Scardino et al., 2009; Graham & Cady, 2014). Moreover, the vast majority of the studies available neglect the results of the biofouling effect which is present *in vivo* and significantly hinders surface activity designed *in vitro* in the laboratory environment. For example, in a dynamic biological environment such as the oral cavity, the implant surface will always be covered by a protein and sugar-rich pellicle, which may cover a certain nanotopography and impair its contact-killing abilities. Conversely, by applying antifouling surfaces based on nanoscale-tailoring one may decrease the adsorption of proteins and bacterial settlement but will also decrease attachment of the host’s cells needed for implant integration (Hsiao et al., 2014). Also, the properties of a given surface may differ under static and dynamic conditions, which leads to confusing results between the available studies (Pechook et al., 2015).

**Figure 4.**
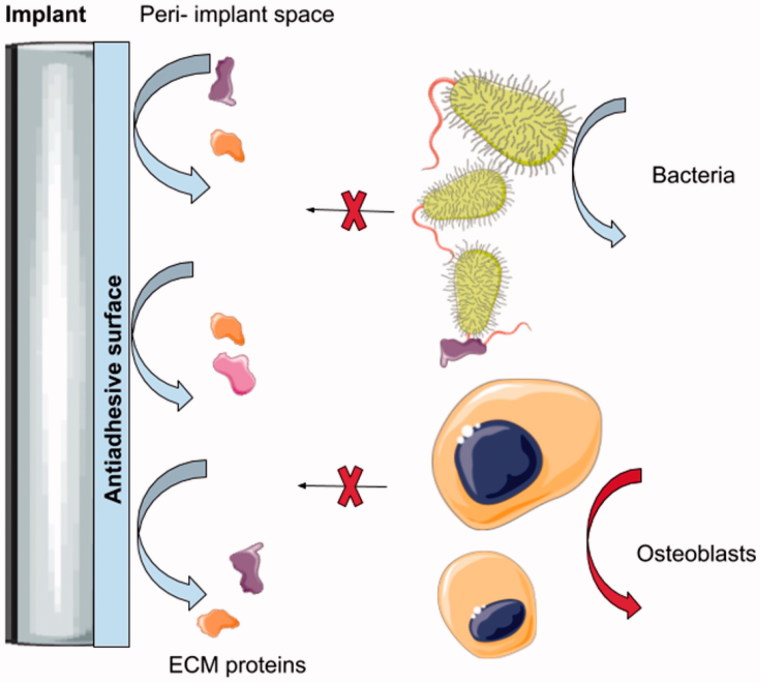
Illustration shows the mechanism of action of the anti-adhesive coating on the intraosseus titanium implant. It may be seen that the repulsing effect inhibits the adsorption of the proteins which are crucial for early-colonizing bacteria settlement. This prevents any subsequent biofilm formation. On the other hand, proteins are required for the pre osteoblast cells and proper osseointegration of the device within the bony tissue. Therefore, antiadhesive surfaces, despite decreasing biofilm formation, may also affect the integration of the intraosseous devices.

In general, antiadhesive approaches at the nanoscale level are designed to repulse the proteins necessary for bacterial settlement on the surface. However, such coatings are unlikely to be selective and may interfere in the course of osseointegration. As antiadhesive coatings may decrease the adsorption of the proteins required for efficient bonding with bone, it may not be appropriate for materials where bone regeneration is required (Figure 4) (Pogodin et al., 2013). Otherwise, it is worth evaluating such an approach with regard to the soft-tissue piercing elements of type E implants (Figure 1(a)). Antiadhesive coatings may find a practical application in the cases of the percutaneous and permucosal portions of these implants as the site of the peri-implant soft tissue collar constitutes *locus minoris resistntiae* for bacterial leakage and a gateway for any infection spread through the device deep into the bone.

## Antibacterial biomaterials active surface finishing

With regard to intraosseous biomaterials, which require osseointegration, active surface finishing has been more extensively evaluated in recent years. The general principal of the antibacterial strategies was based upon the local delivery of the bactericidal agent into the peri-implant space which, as a consequence, kill bacteria that reach the near proximity of the implant surface (Goodman et al., 2013; Arciola et al., 2015). Such an approach requires certain surface modifications in order to sufficiently incorporate the antibacterial agent, deliver it into the specific anatomical area and provide the necessary pharmacokinetics in the clinical environment (Ranade, 1990). Each drug or substance, which is applied in the body at the right time period, fulfills the definition of ‘local drug delivery’ (Fu & Kao, 2010). Such systems may consist of one or more bio-active agents and excipients that form a medium or vehicle for drug administration. Consequently, titanium biomaterials that dilute drugs or chemical compounds into the peri-implant area should be regarded as drug-delivery systems (DDS), abbreviated here as drug- containing implants (DCIs). Drug delivery into the peri-implant space may be achieved by diffusion, degradation of the coating (e.g. polymer blend) or both. The methodology of drug embedment on the implant surface is closely related to the type of biomaterial of which it is made (solid vs. porous). In the case of solid titanium devices, which represent the vast majority of OMF implants available, drugs may be incorporated onto the surface only (surface delivery). On the other hand, porous implants, which have gained far more attention due to their porosity which enhances osseointegration, may contain drugs on the surface, but also within the pores of the inner structure of an implant as well (bulk delivery) (Huang & Brazel, 2001; Giannakou et al., 2016). In the past 5 years alone, a plethora of research concerning antibacterial implants has been published, however, limited effectiveness or short-term efficacy *in- vivo* remains as an issue (Huang & Brazel, 2001; Giannakou et al., 2016; Joshi et al., 2016; Zhao et al., 2017).

In 1990, Ranade postulated that local drug delivery implants require the increased selectivity of drug action achieved by the system’s zero-order release rate, and a decrease in the frequency of administration (Crommelin & Florence, 2013). Zero-order is the ideal pharmacokinetic response curve. However, in the clinical environment, the release profile usually exhibits multiple extreme peaks and troughs which affect both, antibacterial efficacy and potentially toxic overdosages of drug molecules. Therefore, the clinical applicability of intraosseus DCIs poses a technological challenge (Joshi et al., 2016). The first problem is associated with profiling the substance release rate in a given biological environment (intraoral vs. extraoral). The oral microbiome, hygiene measures, and all patient-related factors may exert an influence on the oral biochemistry and fluid flow at a nanoscale level, which will interfere in different ways with the DCI’s pharmacokinetics tailored *in vitro* to the experimental environment. However, in the vast majority of studies where the implant surface was incorporated with antibacterial agent, excessive and uncontrolled release of the substance from the implant surface in the first hours or days after its insertion into the body was noted. This phenomenon is known as burst release (BR) and is responsible for rapid drug dissolution, which up-regulates its concentration in the peri-implant area. Burst release is a first line factor interfering with the zero-order rule (Chinna Reddy et al., 2011).

The BR is related to the surface characteristics, the type of antibacterial agent, as well as, the methodology of its incorporation into/onto the surface, device geometry, surface characteristics of the host material. There are studies which show that BR may include the first 70% of substance release (Chen et al., 2015). Some authors suggest that BR may be beneficial in potentially contaminated sites, where a high concentration of antibacterial agent is desired over a few days/weeks, e.g. osteomyelitis (Tomasi et al., 2014; Zhao et al., 2017). Overly high concentrations of drugs, may on the other hand induce significant toxicity to the tissues and hence affect the DCI’s biocompatibility requirements. Several approaches have been described so far, which could provide a zero-order drug delivery (Zhang et al., 2016) but most of them are complex, expensive, time consuming, and difficult to manufacture. In addition, such formulation techniques result mainly in a first-order release and none of them refers directly to titanium DCIs devoted to OMFS. Despite the fact that there are plenty of drug delivery systems available for periodontal disease treatment, these take the form of external materials or drugs that are put into the inflamed pocket by the general practitioner and cannot be regarded as DCIs *per se* (Siepmann & Siepmann, 2012).

Another problem, which is integral with DCIs, is the effect of body fluids and their velocity on the pharmacokinetics of drug release. From a clinical point of view, drug-coated implants are characterized by a gradual decrease in the substance release rate into the body fluid. Such phenomena contribute to a decrease in the drug minimal inhibitory concentration (MIC) of the drug/substance and induce the development of bacterial resistance and re-contamination of the implant surface (Ranade, 1990). Such a ‘vicious circle’ of biofilm infections are most probable for permanent biomaterials, especially dental implants which are constantly coated by saliva and bacteria. Additionally, calculating the pharmacokinetics around dental implants is troublesome, as no mathematical model has been developed to quantify drug distribution in the peri-implant space to date.

### Drug delivery from the surface to the peri-implant tissues in the head and neck area – a gap to fill.

Defining local-pharmacokinetics has been an aim for decades, but still, predictable targeting of quantitative delivery to an individual anatomical space has not been achieved to date (Heller, 1987). The unwanted interactions between ligand and ligand-bearing nanoparticles with cell receptors, protein corona formation, conformational changes, cellular phagocytosis, and colloidal aggregation are obvious, and may contribute to a decrease in the antibacterial activity of the substance in the peri-implant space. The factors influencing drug release kinetics are related to the material matrix (composition, structure, swelling degradation), release medium (pH, temperature, ionic strength, enzymes), and drug compounds (solubility, stability, charges, interaction with the matrix) (Chinna Reddy et al., 2011). Therefore, to appropriately tailor DCIs, one must take into account the mechanisms that influence substance dissolution and diffusion within the given tissues, e.g. alveolar mucosa and alveolar bone and or bone, muscle, fascia, and skin (Figure 1). Peri-implant mucositis or cellulitis is a state, where the oral mucosa/skin surrounding an implant becomes inflamed due to bacterial infiltration. Hence, a prediction model that takes into account the soft tissue biochemistry for both the healthy and inflamed state is mandatory (Heller, 1987). To deliver a sufficient amount of drug into the soft tissue, it must overcome the thickness of the gingival epithelium, the mucosal/skin permeability barrier properties and the continuous secretion of saliva (0.5–2 L/day) or sweat leading to a subsequent dilution of the drug from the peri-implant space and enzymatic degradation (Alencastre et al., 2016; Weiser & Saltzman, 2014). It must be noted that mucosa of the alveolar gingiva, which is a part of the masticatory mucosa differs from the buccal-mucosa, with which, most of the DCIs were investigated (Nicholson, 1995; Alencastre et al., 2016). Gingival mucosa is more dense and less permeable due to the surface epithelial layer and a deeper connective tissue layer (*lamina propria*) which is compact fibrous tissue, comprising two collagen-rich sub-layers providing a firm connection to the bone, with a function of resisting compression and shear forces (Holpuch et al., 2010). Moreover, it must be noted, that the cytoarchitectonics of the peri-implant mucosa differs significantly from the gingiva of the natural teeth. It is characterized by scar nature (low percentage of fibroblasts, irregulars collagen fibers structure), and a lack of periodontal space (diminished blood and interstitial blood flow), which decreases its permeability (Zhang et al., 2012; Jin et al., 2017).

The mucosal permeability is related to its viscosity and elasticity, hence it will be affected by different mechanical conditions, decreasing or increasing the flow during implant functioning (Holpuch et al., 2010). This is reasonable, as a properly healed implant must be immobile, because osseointegration is a kind of functional ankylosis. If the implant is even slightly mobile it enhances the bacterial leakage at the implant-gingival interface seal, resulting in bacterial infection. Mucosal permeability is significantly reduced during an inflammation due to increased hydrostatic pressure (the normal HP of the gingiva is between 1.14 and 1.23 kP), which, by reducing blood flow generates ischemia and decreases the interstitial flow (Holpuch et al., 2010).

Passive diffusion is generally considered to be the main mechanism by which drugs migrate from the implant surface into the tissues. Based on the studies of drug diffusion through the oral mucosa, it is clear that currently there are no models defining precisely the mechanism of internal drug passage (parallel to the mucosa) along with interstitial fluid. The vast majority of the studies focus on drug delivery from the external environment (perpendicular to the mucosa), and these are usually based on Fick’s first law (equation defining concentration under the assumption of steady state) and Fick’s second law (predicts how diffusion causes concentration to change over time) which simplifies the oral mucosa to a hydrophobic, semipermeable membrane (Holpuch et al., 2010; Ouyang et al., 2018) and hence, these are frequently applied to mathematical models describing drug absorption through such membranes (Ohri et al., 2013).

A comprehensive presentation of mathematical models available, which may be applied in order to predict drug release from particular types of dosage forms of implants was published by Siepmann & Siepmann (2012) (Santamaria et al., 2017). As a result of their work, it may be concluded that dental implants covered with antibacterial agent are most likely to act as monolithic systems, with the drug and the release rate controlling material homogeneously distributed throughout the device. Monolithic systems may release the substance by diffusion, swelling, or erosion (Romagnoli et al., 2013). Hence, Fick’s second law of diffusion may be helpful for cylinders, such as oral implants, allowing for the calculation of the cumulative amount of drug released as a function of time *t*:

$$\frac{M_{t}}{M_{\infty }}=1-\frac{32}{\pi^{2}}\sum_{n=1}^{\infty }\frac{1}{q_{n}^{2}}\text{exp}\;\left(-\frac{q_{n}^{2}}{R^{2}}Dt\right)\cdot \sum_{p=0}^{\infty }\frac{1}{{\left(2p+1\right)}^{2}}\cdot \;\text{exp}\;\left(-\frac{{\left(2p+1\right)}^{2}\pi^{2}}{H^{2}}Dt\right)$$

where *M_t_* and *M*_∞_ denote the cumulative amounts of drug released at time *t* and at infinite time, respectively; *D* is the diffusion coefficient of the drug within the system, and *R* and *H* represent the radius and height of the cylinder, respectively (Santamaria et al., 2017). When the drug is homogeneously distributed within a matrix at an initial concentration that exceeds drug solubility and comes into contact with aqueous body fluids, water penetrates into the system and only partially dissolves, the drug-device should be regarded as a monolithic dispersion system where the Higuchi equation is proposed:

$$M_{t}=A\sqrt{Dc_{s}\left(2c_{ini}-c_{s}\right)\cdot t}$$

where *M_t_* denotes the cumulative absolute amount of drug released at time *t*; *A* is the total surface area of the film exposed to the release medium (Santamaria et al., 2017). Based on the aforementioned models, drug release from the device is usually based on drug diffusion due to concentration gradients. The main problem with the simplified models for drug release is that they disregard interactions of the active molecules with the environment and consider only the pseudo steady-state, making *in silico* prediction inaccurate in the clinical reality (Ohri et al., 2013; Garg & Goyal, 2014). The dynamics of local transport of the tissues govern the spatial pattern of drug distribution, regardless of the design of the delivery system (Moioli et al., 2007). Release of a substance from the device into a tissue, will proceed within its three phases: intracellular space (ICS), a cellular membrane (CM), and an extracellular space (ECS) (Moioli et al., 2007). The ratio of each differs between the tissues and this leads to the heterogeneity of spatial variability of the drug along the implant surface. Different tissues/organs require separate models for drug spatial pattern distribution from the given DCI (Priya James et al., 2014; Bose et al., 2018). For example, as an oral implant is embedded in both, bone and connective tissue, it seems that the total concentration and spatial distribution require separate modeling for: implant-mucosa, implant-bone, bone-mucosa interfaces, and drug diffusion (Figure 1).

There are studies indicating that surface incorporation with different antimicrobial agents provides sustained drug release for up to several weeks (Ranade, 1990). In previous chapters the issues with defining the concentration of the drug in the peri-implant and factors influencing its diffusion and spatial distribution space were discussed. In the case of antibacterial DCIs, the most important factor is to provide a drug concentration (*C*), i.e. capable of providing bactericidal properties (Minimal Inhibitory Concentration – MIC). In 2012, Dukhin and Labib formulated the ‘theory of the local effective release’ which was based on the Higuchi model for the optimization of the polymer–drug blend used as implants (Ranade, 1990). In their model, the ‘effective release concentration’ definition was used for the first time with regard to DCIs providing sufficient MIC and was based on the Higuchi invariant and the characteristics of convective diffusion within the given tissue and will be discussed below.

**Figure 5.**
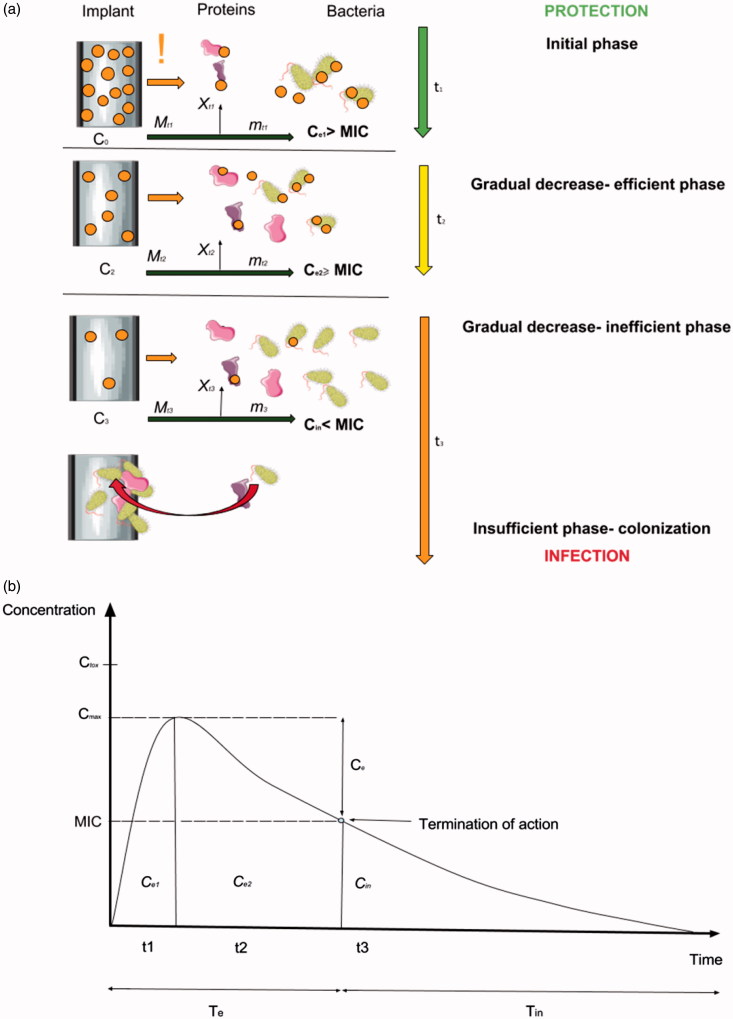
The graph showing the longevity of the drug-releasing implant shown as a scenario ‘from protection to infection’ (a) and peri-implant drug release time profile (b) where burst release (*t*_1_) and gradual decrease in its efficient period (*t*_2_) stand for the effective release time period (*Te*) providing antibacterial properties (*C_e_* > MIC) (protection). Constant release of a drug from the implant surface during its functioning in the clinical environment and the dynamic flow of biological fluids result in an inevitable decrease in effective drug release. This begins as a period of ineffective release (*T_in_*), where the implant still releases some portions of the drug, but it is insufficient to inhibit bacterial growth (C*_in_*< MIC), and moreover, it may contribute to bacterial resistance development. If the implant is left within the tissues, it may become recolonized by bacteria and induce infection development (insufficient phase).

According to the current state-of-the-art, there is no ideal biomaterial that could fulfill all the requirements for intraosseous DCIs (Joshi et al., 2016). Therefore, in order to better understand the clinical requirements for the DCIs for OMFS, the hypothetical longevity of such a biomaterial was divided into three phases with the substance release profile seen in vast majority of the studies published on DCIs (Figure 5). 

It may be seen soon after the implant insertion into the bone, that the excess of the antibacterial agent is being released into the peri-implant area (marked as ‘!’) (Figure 5). The procedure of insertion and mechanical disruption of the outer layer of the surface, as well as, the release of the less-stable particles of the antibacterial agent contribute to the BR. The phenomena of BR was not extensively evaluated and, surprisingly, ignored in most available mathematical models due to its short time scale compared to the entire release process, and hence, its negligible effect. Currently, it is well established that BR is a significant part of DCIs and is a considerable variable modifying release-rate, interferes in zero-order release, and may reduce the effective lifetime of the device (Tomasi et al., 2014). The total amount of a drug released during BR may be calculated by equation:

$$M_{t}=\frac{DC_{o}}{l}\left(t+\frac{l^{2}}{6D}\right)$$

where, *D* is the drug diffusion coefficient, *C*_0_ is the drug concentration at inside of the membrane (coating), and *l* is the membrane (coating) thickness and *t* is time.

However, after drug release, the proportion that will interact with the host’s fluids and enzymes is negligible in the context of effective concentration. Therefore, the concentration of the antibacterial agent in the peri-implant space that provides sufficient antibacterial effect (*C_e_*) is calculated:

(I) $$C_{e1}=C_{0}\;–\;(m_{t1}+X_{t1})=C_{0}\;-\;M_{t1}$$

where, *C*_*e*1_ is the functional concentration during burst release (Phase 1), *C*_0_ is the drug concentration on the inside of the membrane (coating), *m*_*t*1_ is the concentration of the drug that interacts with bacteria, *X_t_*_1_ is the proportion of the drug inactivated by the host’s fluids and enzymes in a given environment. It must be emphasized that both, *m_t_*_1_ and *X_t_*_1_ are related to the type of the antibiotic/substance used.

*C_e_*_1_ should exert no toxic effect to human tissues (*C_tox_*)_._ BR is usually observed in the first hours and days after implant insertion. Its duration (*t*_1_) will usually be shorter than the time needed for implant osseointegration (*T_int_*) which is estimated to be 3–6 months and definitely shorter than potential implant functioning thereafter (*T_fun_*). Therefore, Phase I which is most likely to be observed in the case of all titanium made DCIs will follow the conditions below:

(1) $$C_{e_{1}}>\text{MIC}$$

(2) $$C_{e_{1}}<C_{\text{tox}}$$

(3) $$t_{1}<T_{int}$$

(4) $$t_{1\;}<T_{fun}$$

After BR suppression, it would be most desirable to achieve a zero-order release. However, the gradual decrease in the drug release rate is usually observed in most studies available. The period of DCI functioning with the gradual release of the antibacterial agent into the peri- implant still provides sufficient antibacterial effect (>MIC) (Phase 2) and is described as *C_e_*_2_ which is calculated as:

(II) $$C_{e2}=\left(C_{0}–C_{e1}\right)-(m_{t2}+X_{t2})$$

$$=C_{2}–M_{t2}$$

where, *C_e_*_2_ is the functional concentration of the drug on the implant surface, *M_t_*_2_ is the amount of the antibacterial agent released from the surface during Phase II. This period is the most crucial one with respect to both implant healing and infection eradication. Sustained, sufficient drug release into the peri-implant should cover the most vulnerable period when the implant is not fully integrated, yet is still, under increased risk of bacterial contamination (*T_int_*). When the implant is integrated, its functional exploitation begins (*T_fun_*). Sometimes, *T_int_* overlaps with *T_fun._* (Table 1). At this point it is necessary to incorporate the *T_e_* value described by Dukhin and Labib (2012), which is described as the time of effective release of the agent from the implant that provides sustained MIC:

(III) $$T_{e}=\frac{\text{DCsCo}}{2{\left(MIC\right)}^{2}}{\left(\frac{\delta }{Dti}\right)}^{2}$$

where, *C*_0_ is the concentration of the dispersed drug, *D_ti_*is the diffusivity within the tissue, *D* is the drug diffusion coefficient, and *C_s_* is the solubility limit for the drug dissolved in the polymer/coating and diffusion layer thickness *δ*, which is controlled by the velocity of the interstitial fluid in tissue (Ranade, 1990). However, according to Equation (II) the concentration of the drug at the implant surface after BR from which Phase II begins is *C_f_*_2_, therefore:(IV)

$$Te_{2}=\frac{\text{DCsCe}2}{2{\left(MIC\right)}^{2}}{\left(\frac{\delta }{Dti}\right)}^{2}$$

Drug release should be constant to reduce the risk of dose dumping. From a clinical point of view, Phase II should provide antibacterial properties throughout implant functionality and should also satisfy the following conditions:

(5) $$C_{e2}>\text{MIC}$$

(6) $$C_{e2}<C_{tox}$$

(7) $$\sum_{t2,\;t1}\geq T_{int}$$

(8) $$\sum_{t2,\;t1}\geq T_{fun}$$

(9) $$T_{e}>T_{int}$$

(10) $$\;T_{e}\geq T_{fun}$$

After gradual substance release, the concentration in the tissues will inevitably reach the boundary level for the MIC value. Then, DCI will reach the ‘inefficient phase’ (Phase III) when the device is still releasing the substance into the tissues, yet its concentration in the peri-implant tissue is at sub-inhibitory level or below the MIC value. The concentration of the antibacterial agent in the peri-implant that does not provide a sufficient antibacterial effect (<MIC) is described as *C_in_*_._ At this time, it expected that the implant is at least fully integrated (*T_int_*) or that its functional exploitation is ongoing (*T_fun_*). This phase may be described by the following conditions:

(11) $$\;C_{in}<\text{MIC}$$

(12) $$C_{in}<C_{tox}$$

(13) $$\;\sum_{t2,\;t1,\;t3}<T_{int}$$

(14) $$\;\sum_{t2,\;t1,\;t3}<T_{fun}$$

(15) $$\;Te<T_{int}$$

(16) $$\;Te\;<T_{fun}$$

If the implant that has lost its antibacterial activity is left within the tissues, it may be at risk of bacterial re-colonization and infection development.

From a clinical perspective, the most important part, Phase II should last as long as the implant is necessary in order to achieve treatment success (*T_fun_*). Therefore, it is desirable to provide *T_e_*> *T_int_*and *T_e_*> *T_fun_*. This may vary between the types of implants and indications for their use. For example, both, extra and intraoral distractors that are used for jaw distraction are usually kept within the body tissue for 3–4 months. After sufficient bone distraction they are removed, and therefore, functional concentration (*C_e_*) through *T_int_* and *T_fun_* is relatively achievable and desired in most intraosseous metallic biomaterials (Joshi et al., 2016). Conditions (9) and (10) are achievable when *T_int_ = T_fun._*This is not the case with dental implants, which require a minimum of 3 to 6 months to integrate with bone and after that period are functionally loaded with prosthetic superstructures. This entity is unique, as in the case of dental implants, infection development is much likely to occur after *T_int_*- and during *T_fun_*. According to current standards in oral implantology, *T_fun_* for dental implants should be at least 10 years. It is unlikely that any type of antibacterial coating would provide such prolonged *C_e_*and satisfy condition (10), when *T_fun_* is significantly longer than *T_int_*_._ Therefore, intraosseous implants designed to be oral and maxillofacial implants should ideally aim at the following derivation to find practical application in the clinical environment:

(V) $$C_{fTe}=C_{o}–(m_{Te}+X_{Te})=C_{o}–M_{Te}$$

and follow the condition:

(17) $$T_{e}\geq \sum_{\text{Tfunc},\;\text{Tint}}$$

where, *T_e_*is the time of effective release, *C*_f_ *_Te_*is the concentration of the drug in the peri-implant space that provides > MIC, *C_o_* is the drug concentration at the inside of the membrane (coating), *M_Te_*is the total drug concentration that diffused from the implant surface during *T_e,_ m_Te_*is the concentration of the drug that interacts with bacteria, and *X_Te_*is the concentration of the drug being inactivated by host proteins and enzymes in the given peri-implant space.

## Conclusions and future research outlook

Both, antifouling and antibacterial surfaces which facilitate local drug delivery have advantages and drawbacks. Antifouling surfaces, by decreasing proteins may inhibit bacterial attachment but simultaneously interfere in osseointegration and should not be applied in intraosseous implants. However, such an approach is likely to be applicable with regard to permucosal and percutaneous parts of the implant in order to decrease pin-site infections. Antibacterial surfaces working through local drug delivery are of great interest in the case of bone-embedded implants. Nevertheless, recently developed biocidal-releasing materials proved to exhibit only short-term efficiency due to the limited amount of the antibacterial compound and resistance to the drug developed by the bacteria. Therefore, new approaches to local delivery are required in overcoming limitations derived from the mathematical models available (Heller, 1987; Moioli et al., 2007; Tappa & Jammalamadaka, 2018). Designing materials providing antibacterial local drug delivery especially for intraosseous, partially external implants requires multifactorial analysis in evaluating the spatial and temporal pattern of drug concentration through both: hard and soft tissues into the peri-implant space. Moreover, defining the exact period of the desired antibacterial properties and providing sufficient concentrations of the drug by the determination of local cellular pharmacokinetic and regio-specific kinetics is a priority in order to move away from empiricism. The proposed classification of OMF implants and derivations may serve as practical, clinically based guidelines for designing new biomaterials. The vast majority of temporary intraosseous OMF implants require a 3 to 6-month period of sustained drug release. After this period, the devices are removed and infection due to surface re-contamination is unlikely to occur.

In the case of dental implants, currently available techniques are unlikely to provide an antibacterial effect through local drug delivery, as dental screws are permanent implants which are expected to function flawlessly for at least 10 years after implantation.

Modifications providing effective drug release throughout this period into the peri-implant space are possible via the structural design of the surface at a nanoscale level and/or the application of biodegradable or non-biodegradable polymers and composites that may enhance sustained antibacterial activity and decrease the inflammatory response (Campoccia et al., 2013; Zhao et al., 2014; Elter et al., 2016). As an example, polylactide acid (PDLA) nano-formulations were shown to increase the duration of drug release more than 20-fold in the study by (Gadenne et al., 2013; Ohri et al. 2013). Other formulations discussed by Santamaria et al. (2017), that may provide prolonged drug release were hydrogels and hybrid formulations, such as: polymeric particle-hydrogels or liposome-hydrogels (López & Blázquez, 2009). Polymer-based scaffolds are a group of biomaterials that may provide a multifactorial impact on the surrounding tissues. They may be applied as both, a tissue regenerative material and a drug delivery system loaded with the clinically-desired agent (Laffleur & Bernkop-Schnürch, 2013). The advantages of scaffolds as drug delivery systems are as follows: high loading efficiency and surface area; stability as well as control over content release (Lindert & Breitkreutz, 2017). The use of scaffolds is still challenging in the CMF area due to the complex environment and the necessity to accommodate multiple tissue phenotypes (Horváth et al., 2013). Moreover, despite immense progress in biomaterials engineering, polymeric drug delivery systems have not been used in standardized clinical applications to date (Zhang et al., 2002).

Additional solutions could be achieved by the implementation of 3D customized implants (also known as additive manufacturing) with desired properties, adjusted to the given clinical requirements (Mokhtarzadeh et al., 2017). As stated by Bose et al. (2018) and Palo et al. (2017) such an approach could be helpful in the improvement of patient-specific therapy and could also overcome the limitations of current local drug delivery systems (Maher et al. 2017; Mokhtarzadeh et al., 2017; Palo et al., 2017). already described customized localized anticancer drug-releasing titanium implants with prolonged drug release and sufficient implant osseointegration Maher et al. (2017). As 3D printing technologies are undoubtedly going to expand the medical market, the personalization of drug delivery systems and the anatomical modeling of CMF implants is of great interest (Tappa & Jammalamadaka, 2018). Solutions with bio-sensing properties and a stimuli-response based on pH and ionic species and/or biodegradable polymer drug delivery systems which release the desired amount of substance when necessary are also required for such a purpose. Such an approach requires further investigation and more pre-clinical evaluation.
